# Autoimmune enteropathy in an infant, a rare entity possibly triggered in utero

**DOI:** 10.1093/omcr/omaf186

**Published:** 2025-09-28

**Authors:** Sava Grujic, George Gershman

**Affiliations:** Department of Pathology, Harbor UCLA Medical Center, 1000 W Carson St, Torrance, CA 90502, USA; Department of Pediatrics, Harbor UCLA Medical Center, 1000 W Carson St, Torrance, CA 90502, USA

**Keywords:** autoimmune enteropathy, infant watery diarrhhea

## Abstract

Autoimmune enteropathy is a rare immune mediated disorder with incidence of less than 1 in 100 000 that primarily involves infants and children. It characterized by severe and protracted diarrhea, weight loss and immune-mediated damage to the intestinal mucosa. We report a case of previously healthy infant that developed acute diarrhea at 7 weeks with a large volume of watery stool. A trial with amino acids-based formula was unsuccessful. Biopsies taken during esophagogastroduodenoscopy and ileo-colonoscopy performed at 8 weeks were consistent with autoimmune enteropathy. Treatment with intravenous steroid and Sirolimus was initiated with an excellent response. At 16-week follow-up the child was doing well without need for immunosuppression. Neonatal immune system is naïve with scant plasma cells normally found at this age. Considering the presence of numerous of plasma cells in the biopsy material, both IgM and IgG class, we postulate that this process was possibly triggered in utero.

## Introduction

Autoimmune enteropathy (AIE) is a rare intestinal disease characterized by immune-mediated intestinal mucosal damage [1]. The incidence of AIE is less than 1 per 100 000 children. Clinical manifestations of AIE include intractable watery diarrhea, growth retardation and hypoproteinemia.

In autoimmune enteropathy, aberrant expression of self-antigens on epithelial cells triggers CD4+ T lymphocytes, which produces downstream effects leading to the destruction of the enterocytes by means of apoptosis or other cytotoxic effects [2].

Autoantibodies directed against intestinal epithelial cells have also been found in the pathogenesis of autoimmune enteropathy and can be directed toward goblet cells, enterocytes, and the intestinal brush border. Autoantibodies directed against AE75, an intestinal antigen that exerts an important function in the integrity of tight junctions and cytoskeleton integrity, lead to enhanced intestinal permeability.

AIE has no specific histological features; however, all patients have gastrointestinal mucosal injuries.

The pathological findings in the duodenum can be divided into four types: 1-active chronic duodenitis expressed by infiltration of the lamina propria with plasma cells predominantly and neutrophilic cryptitis; 2-celiac disease-like with villous blunting and marked increased in intraepithelial lymphocytes in surface epithelium; 3- graft-versus-host disease-like: increased apoptosis in crypt epithelium, and 4-mixed mode [3]. Loss of Paneth and goblet cells is common.

A concomitant gastritis and colitis are present in at least 2/3 of AIE cases. [2].

Serum anti-intestinal epithelial antibodies, anti-enterocyte (AE) or anti-goblet cell (AG) antibodies can support the diagnosis; however, an absence of these antibodies cannot exclude AIE.

Variations in age of AIE onset and diversity of immunological response which translated into the 4 distinct histological patterns of AIE rase the question about the time of triggering event and its potential occurrence in utero in same cases.

## Case report

Previously healthy neonate developed acute diarrhea at 7 weeks of age with a large volume of watery stool without gross blood or mucus. The patient was a product of a healthy pregnancy and a normal spontaneous vaginal delivery. The baby was fed with a combination of breast milk and cow’s milk protein-based formula. The family history was positive for lactose intolerance, eczema, and asthma. Three days after the onset of diarrhea the baby was admitted with hypovolemic shock requiring multiple boluses of normal saline, leukocytosis and hypoglycemia. The patient was never febrile. Work up for infectious etiology of diarrhea including testing for Giardia, Cryptosporidium, Norovirus, Coronavirus, Rhinovirus/Enterovirus was negative. Stool cultures and workup for underlying immunodeficiency were negative.

The whole exome sequencing genetic testing was unremarkable except a biallelic pathogenic variant in *GALE* (GALE (NM_000403.4): c.151C > T, p.Arg51Trp, heterozygous). Biallelic pathogenic variants can be associated with galactose epimerase deficiency and syndromic thrombocytopenia but as heterozygous the patient is unlikely to be affected. There is no known association of these variants and known immunological or gastrointestinal disorders.

Diarrhea persisted despite a 24-hour bowel rest and a trial with Pedialyte and amino acid-based formula. A total parenteral nutrition was started. Esophagogastroduodenoscopy and colonoscopy with multiple biopsies were performed at 8 weeks of age. Anti-enterocyte antibody test was negative.

**Figure 1:**
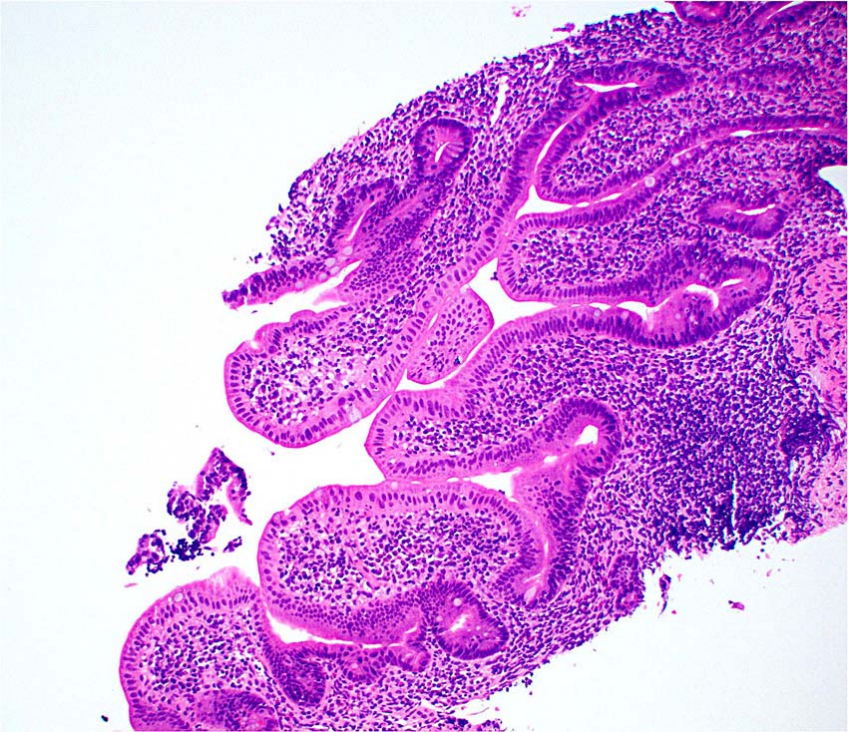
Low magnification image of duodenal biopsy shows villi distended by numerous plasma cells. Goblet cells and Paneth cells are significantly decreased in number.

**Figure 2:**
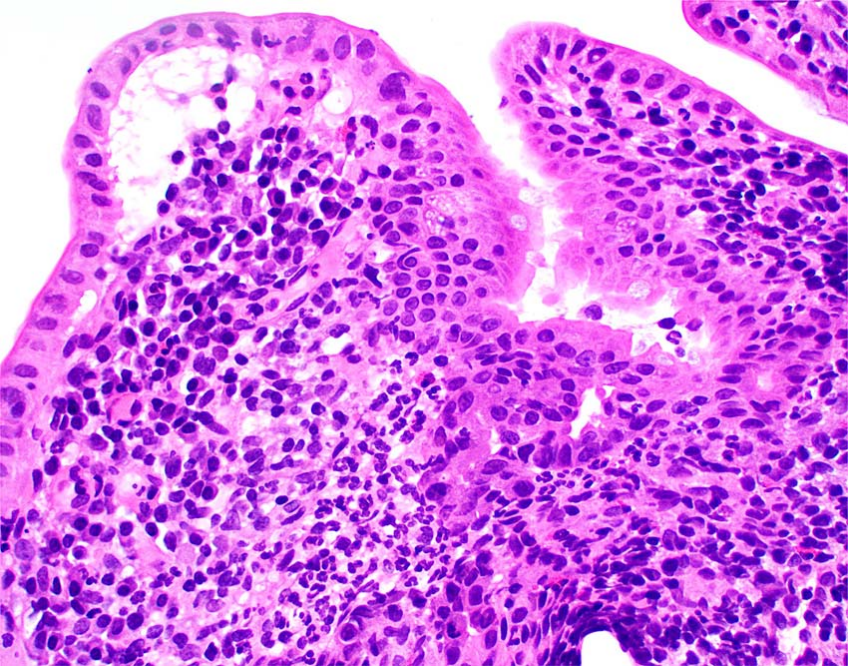
High magnification image of duodenal biopsy demonstrates numerous plasma cells within villi and foci of active inflammation. Intraepithelial lymphocytes are also present.

**Figure 3:**
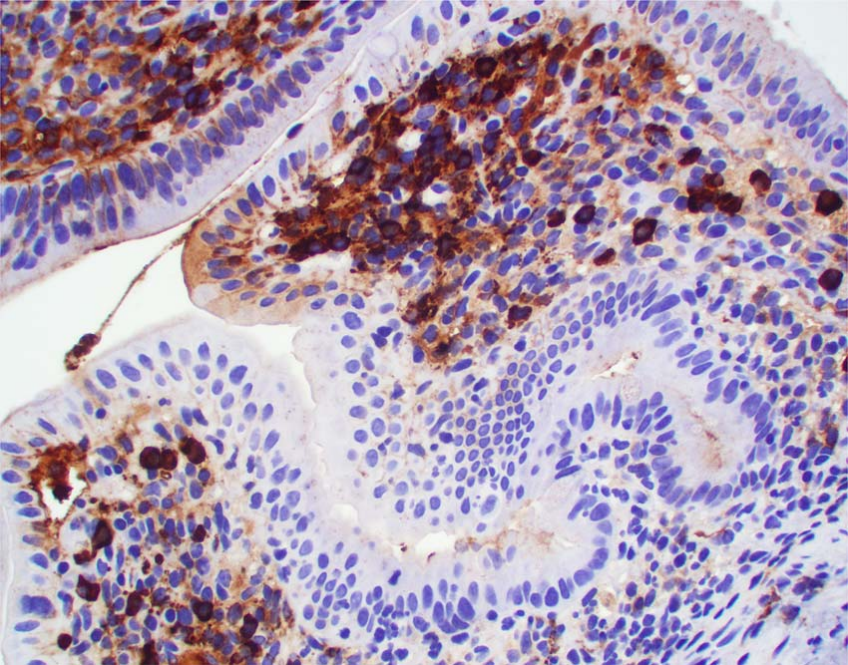
IgM positive plasma cells were predominant. Positive IgM staining is also focally present in the area of tight junctions.

**Figure 4:**
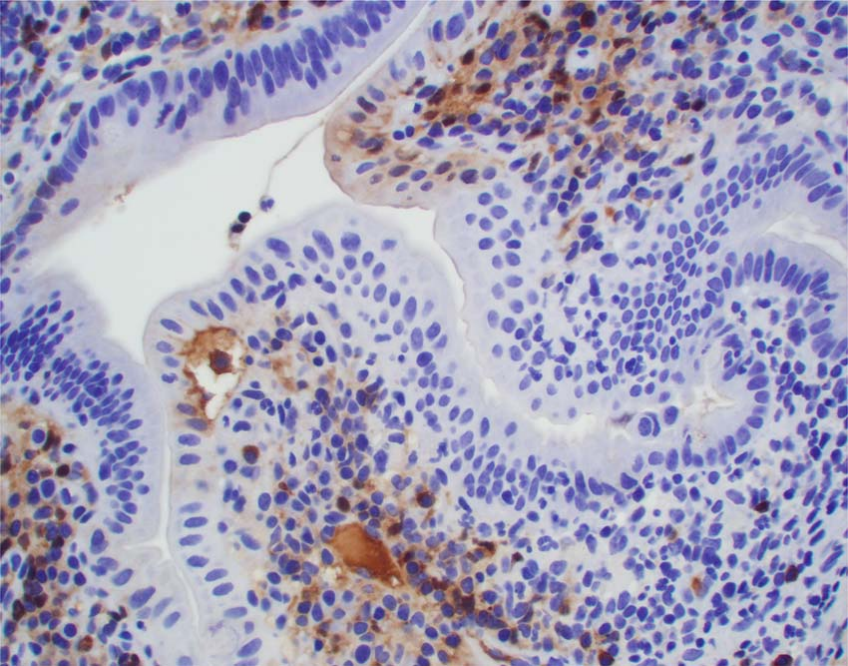
Significant number of IgG positive plasma cells is also present. Positive IgG staining is focally present in the area of tight junctions.

**Figure 5:**
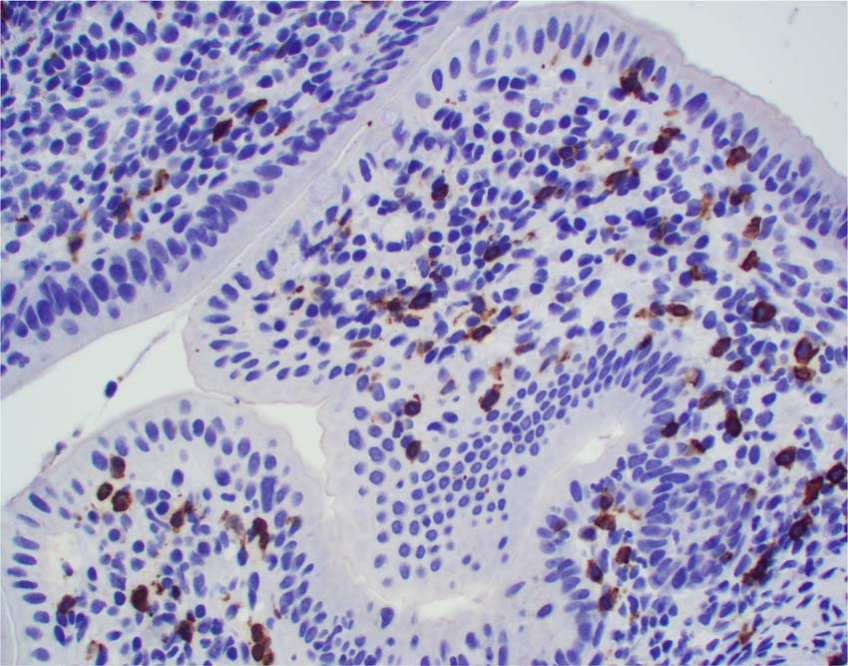
Intraepithelial CD8 cytotoxic lymphocytes highlighted by immunostaining.

**Figure 6:**
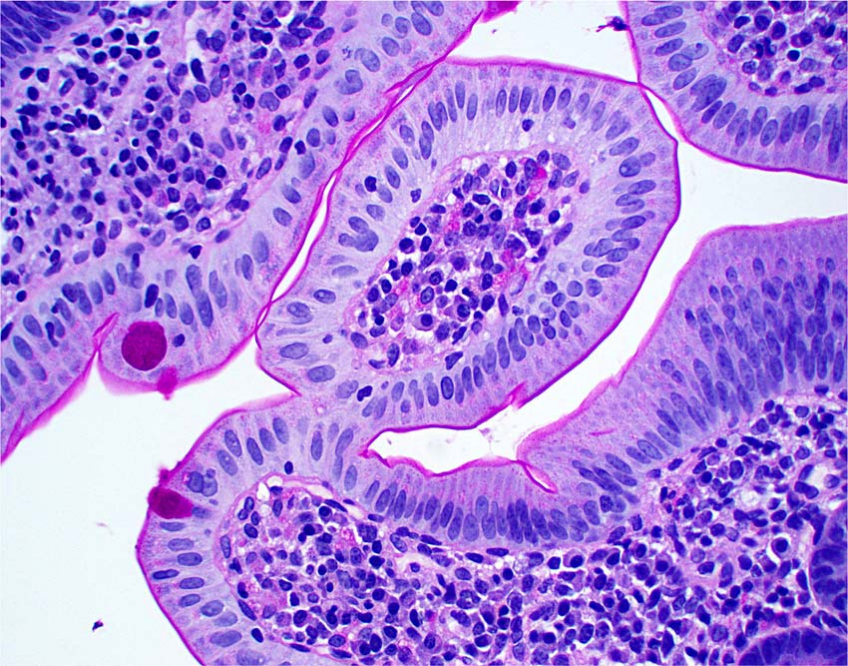
PAS stain of duodenal mucosa highlights intact brush border and only two goblet cells per more than hundred enterocytes. Normal ratio in duodenum is 1 to 5.

Duodenal biopsy (Fig. 1) showed villi distended by numerous plasma cells. Plasma cells are usually scant at this age due to the limited exposure to antigens. IgM plasma cells were predominant (Fig. 3) but a significant number of IgG plasma cells was also noted (Fig. 4). Goblet cells and Paneth cells were significantly decreased in number (Fig. 2). Foci of active inflammation and scattered cytotoxic CD8 positive intraepithelial lymphocytes (Fig. 5) were also present. Brush border was intact, confirmed by PAS stain (Fig. 6) arguing against microvillous inclusion disease. Tufting of the enterocytes was not present excluding tufting enteropathy.

**Figure 7:**
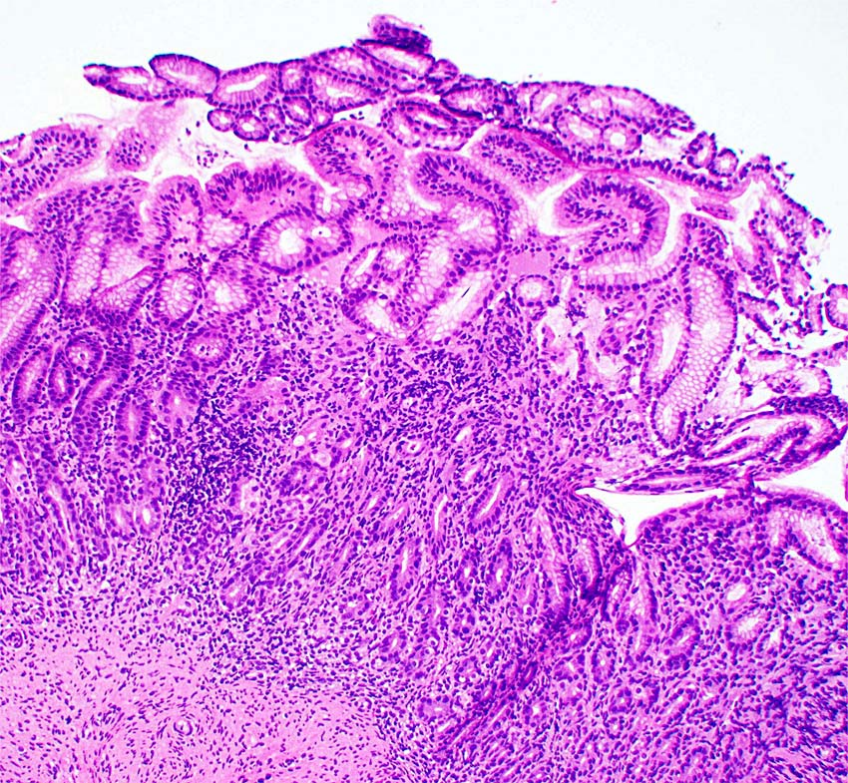
Low magnification image of antral biopsy shows features of chronic gastritis.

**Figure 8:**
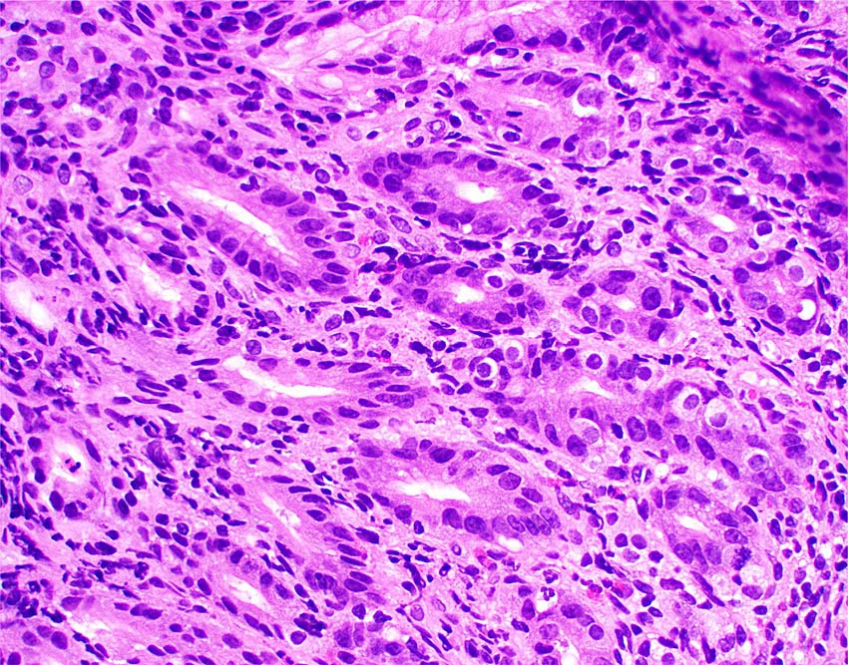
High magnification image of antral biopsy demonstrates intact neuroendocrine, gastrin producing cells with ‘fried egg’ appearance.

**Figure 9:**
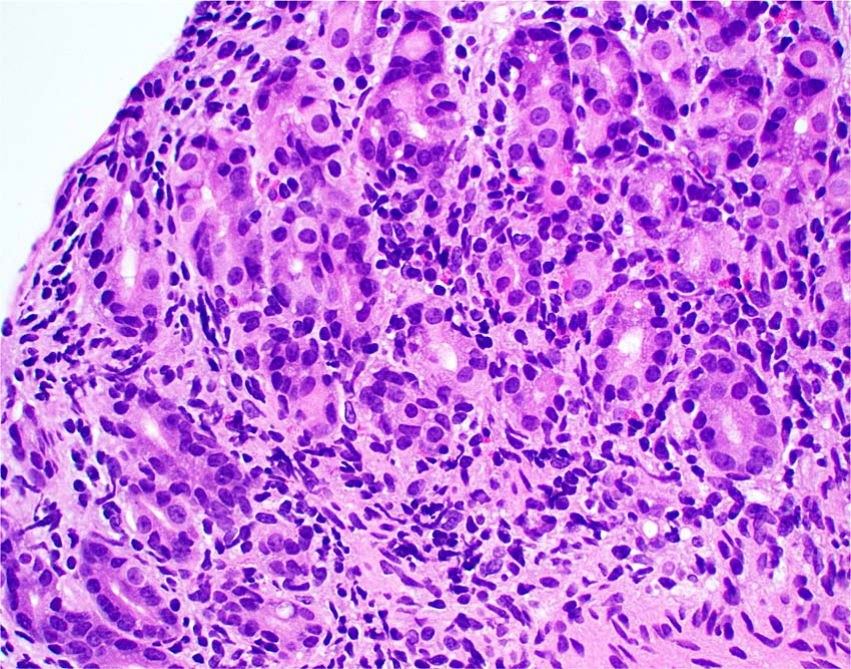
Intermediate magnification image of gastric body biopsy shows atrophy with significantly decreased parietal and chief cells and slightly increased lamina propria eosinophils.

**Figure 10:**
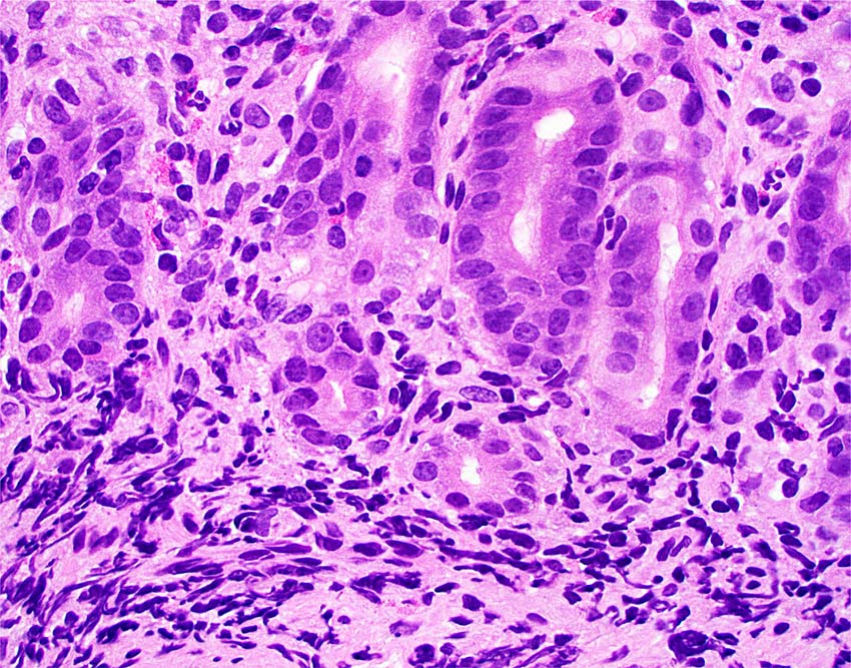
High magnification image of gastric body demonstrates atrophic area with absence of parietal and chief cells.

**Figure 11:**
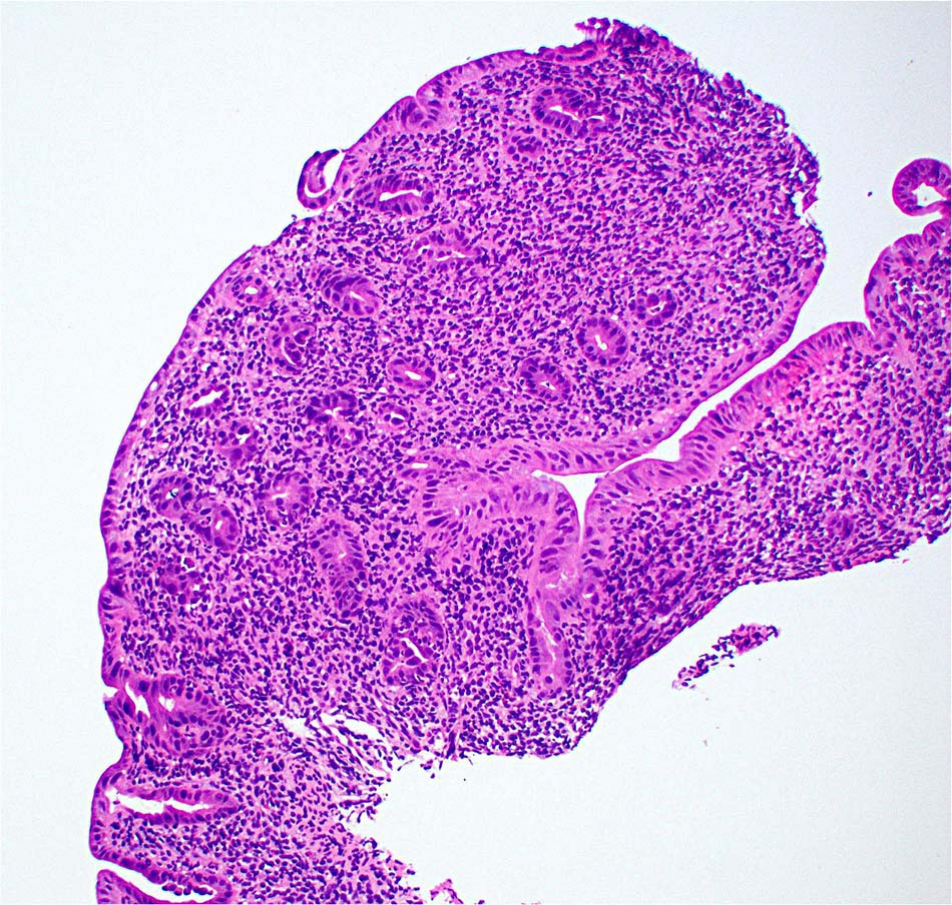
Low magnification image of colon biopsy demonstrates increased lymphoplasmacytic inflammatory infiltrate within lamina propria and decreased goblet cells.

**Figure 12:**
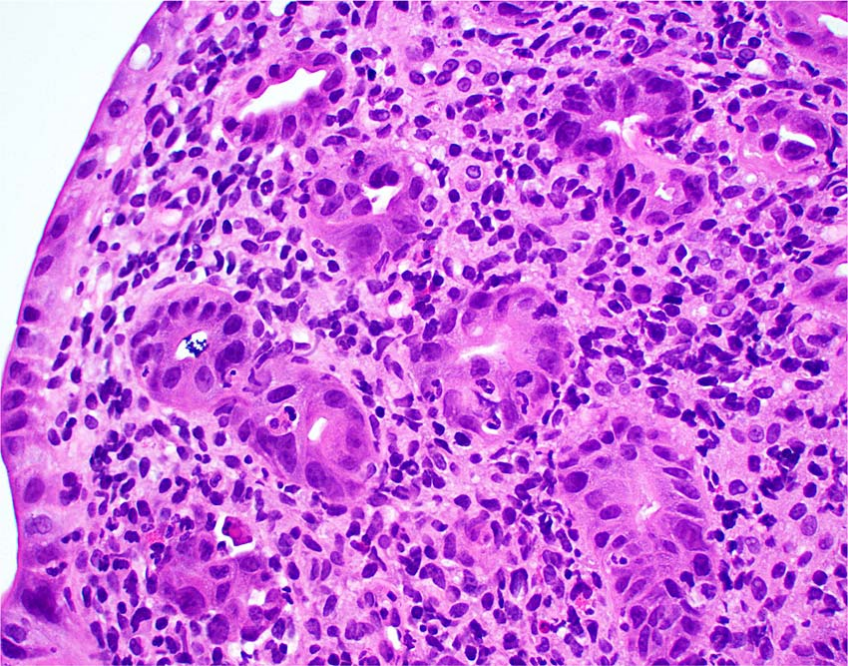
High magnification image of colon biopsy shows multiple foci of cryptitis.

Antral biopsy showed the features of chronic gastritis with significantly increased chronic inflammatory infiltrate within lamina propria (Fig. 7). Neuroendocrine gastrin producing cells with ‘fried egg’ appearance (Fig. 8) were clearly visible arguing against endocrine cell dysgenesis.

Gastric body biopsy showed atrophic changes (Fig. 9) with significantly decreased parietal and chief cells. Increased chronic inflammatory infiltrate was present within lamina propria. Eosinophils were conspicuous (Fig. 10). Intestinal metaplasia was not present.

Colon biopsy showed increased lymphoplasmacytic inflammatory infiltrate within lamina propria and decreased number of goblet cells (Fig. 11). Multiple foci of active inflammation were present (Fig. 12).

After biopsy results that favored AIE, the patient was started on IV steroids 2 mg per kilogram per day for five days and showed significant improvement. Weaning from steroids continued every five days in 0.5 mg BID decrements.

Sirolimus was also started and titrated to appropriate levels. At the time of discharge electrolytes were stable.

Medications were discontinued as planned and the patient was doing well on amino acids-based formula at 16-weeks follow-up visit.

At 5 month visit the child was developing well and had no diarrhea. The only prescribed medications were oral cholecalciferol daily and acetaminophen as needed.

## Discussion

Most patients with AIE are diagnosed as infants during the first few months of life, but cases of AIE have been described in adults.

Genetic alterations underlie two syndromic forms of AIE, namely immune dysregulation, polyendocrinopathy, enteropathy, X-linked (IPEX) syndrome and autoimmune polyendocrinopathy–candidiasis–ectodermal dystrophy (APECED) syndrome [3, 4].

IPEX syndrome is characterized by mutations in the *FOXP3* gene, which cause altered regulatory T cell function, lastly leading to immune overactivity in response to antigen stimulation.

APECED syndrome is due to mutations in the autoimmune regulator (AIRE) gene, which encodes a transcription factor involved in thymic T cell negative selection, resulting in the presence of circulating self-reactive T cells.

In our case without detected genetic alterations the exact mechanism is unknown but it involves both cellular and humoral immune response that probably started in utero. The most likely target are tight junctions [5] that are focally coated by IgM and IgG on our immunostains. The damage of tight junctions would explain abundant watery diarrhea in this patient.

The density of *infant’s plasma* cells in the intestinal lamina propria is increasing progressively over the first two years of life [6]. In our case small intestinal villi were packed with plasma cells in numbers not seen even in normal adults. Both IgM positive plasma cells that represent the primary response and secondary IgG positive plasma cells were present suggesting prolonged process. CD8 positive cytotoxic lymphocytes identified within epithelial lining likely represent cellular component of immune response.

The presence of antibodies against intestinal cells (anti-enterocyte and anti-goblet cell antibodies) is helpful but not required for diagnosis of AIE [7]. Although these antibodies can trigger the complement cascade, their pathogenic significance remains uncertain. It is generally acknowledged that antibodies against intestinal cells represent a late immunologic phenomenon, and the serum titer does not correlate with the severity of the disease [8–10].

The management of AIE is extremely challenging and optimizing nutritional status is essential. Corticosteroids are generally used as first-line therapy and prompt and complete response to steroid treatment in this case confirms the diagnosis. At 5 month visit our patient was doing well and did not require immunosuppression maintenance.
